# Potential role of the methylation of VEGF gene promoter in response to hypoxia in oxygen‐induced retinopathy: beneficial effect of the absence of AQP4

**DOI:** 10.1111/jcmm.13348

**Published:** 2017-09-22

**Authors:** Francesco Pisani, Maurizio Cammalleri, Massimo Dal Monte, Filippo Locri, Maria Grazia Mola, Grazia Paola Nicchia, Antonio Frigeri, Paola Bagnoli, Maria Svelto

**Affiliations:** ^1^ Department of Biosciences, Biotechnologies and Biopharmaceutics University of Bari Bari Italy; ^2^ Department of Biology University of Pisa Pisa Italy; ^3^ Department of Basic Medical Sciences, Neuroscience and Sense Organs Bari Italy; ^4^ Institute of Biomembranes and Bioenergetics National Research Council Bari Italy

**Keywords:** Hypoxia, neovascularization, electroretinogram, HIF‐1 binding site, CpG methylation, AQP4, chromatin immunoprecipitation

## Abstract

Hypoxia‐dependent accumulation of vascular endothelial growth factor (VEGF) plays a major role in retinal diseases characterized by neovessel formation. In this study, we investigated whether the glial water channel Aquaporin‐4 (AQP4) is involved in the hypoxia‐dependent VEGF upregulation in the retina of a mouse model of oxygen‐induced retinopathy (OIR). The expression levels of VEGF, the hypoxia‐inducible factor‐1α (HIF‐1α) and the inducible form of nitric oxide synthase (iNOS), the production of nitric oxide (NO), the methylation status of the HIF‐1 binding site (HBS) in the *VEGF* gene promoter, the binding of HIF‐1α to the HBS, the retinal vascularization and function have been determined in the retina of wild‐type (WT) and AQP4 knock out (KO) mice under hypoxic (OIR) or normoxic conditions. In response to 5 days of hypoxia, WT mice were characterized by (*i*) AQP4 upregulation, (*ii*) increased levels of VEGF, HIF‐1α, iNOS and NO, (*iii*) pathological angiogenesis as determined by engorged retinal tufts and (*iv*) dysfunctional electroretinogram (ERG). AQP4 deletion prevents VEGF, iNOS and NO upregulation in response to hypoxia thus leading to reduced retinal damage although in the presence of high levels of HIF‐1α. In AQP4 KO mice, HBS demethylation in response to the beginning of hypoxia is lower than in WT mice reducing the binding of HIF‐1α to the *VEGF* gene promoter. We conclude that in the absence of AQP4, an impaired HBS demethylation prevents HIF‐1 binding to the *VEGF* gene promoter and the relative *VEGF* transactivation, reducing the VEGF‐induced retinal damage in response to hypoxia.

## Introduction

Aquaporins (AQPs) are integral membrane water channel proteins allowing water to flow through cell plasma membranes in response to osmotic and hydrostatic gradients 1. Among AQPs, AQP4 is the most abundant in brain and retina 2 and its role in maintaining water homoeostasis in the retina has been extensively studied 3. AQP4 has been localized to the Müller cell end feet, where it facilitates water flux at the glial–vascular interface 3, 4. AQP4 deletion results in a gliotic phenotype 5 although this phenotype does not result in altered retinal responses to light 6. There are several indications that AQP4 is overexpressed in experimental models of retinal diseases 7, 8, 9, 10, 11, 12, 13, 14, 15, 16. In some of these models, AQP4 downregulation is generally associated with an amelioration of the pathological signs of the disease 10, 11, 16, although AQP4 silencing has been shown to worsen the pathological signs of diabetic retinopathy 9. These observations indicate that a possible role of AQP4 in retinal diseases deserves attention.

Major diseases of the retina are those characterized by neovessel growth resulting from the accumulation of several proangiogenic and inflammatory factors in response to hypoxia among which VEGF plays a major role 17. In this respect, anti‐VEGF therapies are currently approved to treat proliferative retinopathies such as neovascular age‐related macular degeneration 18.

There are findings indicating the possibility of an interplay between AQP4 and VEGF or that common mechanisms may participate in regulating AQP4 and VEGF expression. Indeed, increased production of both AQP4 and VEGF has been reported in the rodent retina in response to hypoxia or hyperglycaemia 8, 11, 14, 16. In addition, VEGF administration has been found to induce AQP4 expression, whereas decreasing VEGF results in reduced retinal levels of AQP4 11, 16. There is also evidence of functional interactions between AQP4 and hypoxia‐inducible factor‐1 (HIF‐1), the major transcription factor regulating VEGF levels in response to hypoxia 19. For instance, in rat models of cerebral oedema, AQP4 and HIF‐1 are concomitantly upregulated by hypoxia and the inhibition of HIF‐1 prevents AQP4 upregulation 20, 21.

The mouse model of oxygen induced retinopathy (OIR) represents a good model in which to study whether AQP4 may influence VEGF upregulation in response to hypoxia together with its downstream retinal damage 22. In this model, mice are exposed to hyperoxia from postnatal day (PD)7 to PD12 when, after returning to normoxia until PD17, they undergo to relative hypoxia that causes increased activity of HIF‐1 23. HIF‐1 is composed of a constitutively expressed β‐subunit (HIF‐1β) and an oxygen‐regulated α‐subunit (HIF‐1α). Under hypoxic conditions, HIF‐1α is stabilized reaching levels that are determined by a balance in positive and negative feedback mechanisms in which NO production plays an important role 24 through increased levels of the inducible form of NOS (iNOS) 24. After HIF‐1α stabilization, HIF‐1α and HIF‐1β translocate from the cytosol into the nucleus where they form a heterodimer that binds to the HBS localized to the hypoxia response element (HRE) in the promoter of HIF‐1 target genes 25. There is indication that in models of ocular neovascular diseases, the induction of VEGF occurs through the HRE in the *VEGF* gene promoter as HRE deletion prevents the hypoxia‐induced VEGF upregulation 26. In addition, it has been reported that hypoxia‐induced erythropoietin gene expression is dependent on the methylation status of HBS, when HBS is methylated, its binding with HIF‐1 is abolished 27. Finally, the methylation of CpG sites in the *VEGF* gene promoter results in reduced HIF‐1‐driven VEGF expression in the human placenta after preeclampsia and in tongue carcinoma cells in response to hypoxia 28, 29, thus suggesting that something similar might occur in *in vivo* models of hypoxia‐driven neovascular diseases of the retina.

In this study, we investigate the role of AQP4 on hypoxia‐induced VEGF upregulation with the resulting neovessel growth and ERG dysfunction that characterize the OIR model. To this aim, retinal level of AQP4, VEGF, HIF‐1α, iNOS, NO as well as retinal neovascularization and function was determined in OIR models from WT and AQP4 KO mice. Furthermore, the methylation status of the HBS in the *VEGF* gene promoter and the physical interaction between HIF‐1α and the HBS was analysed in both strains under hypoxic conditions.

## Materials and methods

### Animals

AQP4 KO mice with a CD1 genetic background and age‐matched CD1 mice used as WT mice were kindly provided by Dr. Hu (Nanjing Medical University, China). Genotyping was performed on tail DNA using standard protocols. Mice were housed in a regulated environment (23 ± 1°C, 50 ± 5% humidity) with a 12‐hrs light/dark cycle (lights on at 8.00 a.m.), provided with food and water *ad libitum*, and supplied with environmental enrichment materials, such as toys and shelters. This study was carried out in strict accordance with the recommendations in the Guide for the Care and Use of Laboratory Animals of the National Institutes of Health and adheres to the ARVO Statement for the Use of Animals in Ophthalmic and Vision Research. Procedures were carried in compliance with the Italian guidelines for animal care (DL 116/92) and the European Communities Council Directive (86/609/EEC) and were approved by the Committee on the Ethics of Animal Experiments of the Universities of Pisa and Bari. All efforts were made to reduce both animal suffering and the number of animals used. Experiments were performed on a total of 92 mouse pups of both sexes, which were killed at PD17. In some experiments, mice at PD12 (16 animals) were also used. In all experiments, mice were anaesthetized with 4% halothane, killed by cervical dislocation and the eyes were enucleated.

### Mouse model of OIR

In a typical model of OIR, litters of mice pups with their nursing mothers were exposed in an infant incubator to high oxygen concentration (75 ± 2%) between PD7 and PD12 before returning to room air between PD12 and PD17 30. Oxygen was checked twice daily with an oxygen analyzer (Pro‐Custom Elettronica, Milano, Italy). Individual litters were reared in either oxygen or room air (controls). All experiments were performed at the same time of the day to exclude possible circadian influences. The data were collected from both males and females and the results combined, as there was no apparent sex difference.

### Real‐time RT‐PCR

After mice death, retinal tissues were rapidly dissected, snap frozen in liquid nitrogen and stored at −80°C until analysis. Total RNA was extracted (RNeasy Mini Kit; Qiagen, Valencia, CA, USA), purified, resuspended in RNase‐free water and quantified using a fluorometer (Qubit; Invitrogen, Carlsbad, CA, USA). First‐strand cDNA was generated from 1 μg of total RNA (QuantiTect Reverse Transcription Kit; Qiagen). In quantitative real‐time RT‐PCR (qPCR) experiments, five independent samples for each experimental condition, each containing two retinas from two different mice either WT or AQP4 KO, were used. qPCR experiments were performed using a kit (iQ Sybr Green Supermix; Bio‐Rad Laboratories, Hercules, CA, USA), and primer sets were obtained from Primer Bank (AQP4 and VEGF) 31 or RTPrimerDB (Rpl13a) 32. The primer set for VEGF was designed to match the VEGF isoforms expressed in the retina. Forward and reverse primers were chosen to hybridize to unique regions of the appropriate gene sequence. Their sequences are shown in Table 1. Primer amplification efficiency (Opticon Monitor 3 software; Bio‐Rad Laboratories) was close to 100%. Target genes were run concurrently with *Rpl13a*, a stable housekeeping gene in the OIR model 33. Samples were compared using the relative threshold cycle (Ct Method) 34. The increase (x‐fold) was determined relative to a control after normalizing to Rpl13a. All reactions were run in triplicate. After statistical analysis, the data from the different experiments were plotted and averaged in the same graph.

**Table 1:** Sequences of primer sets used in qPCR or in bisulphite sequencing

Use	Gene	Primer sequence
qPCR	*Aqp4*	Forward: 5′‐ AGGCTTCAATTACCCACTGGA ‐3′ Reverse: 5′‐ GTGAGCACCGCTGATGTGA ‐3′
qPCR	*VEGF*	Forward: 5′‐ GCACATAGGAGAGATGAGCTTCC ‐3′ Reverse: 5′‐ CTCCGCTCTGAACAAGGCT ‐3′
qPCR	*Rpl13a*	Forward: 5′‐CCAGGTATACAAGCAGGTGTGCTC‐3′ Reverse: 5′‐CATCATTAGGGCCATCCTGGAC‐3′
Bisulphite sequencing	*VEGF Region 1*	Forward: 5′‐ TTGTTTAGTAGTTGTTTTTTTTTTAGGGTTTTG ‐3′ Reverse: 5′‐ TAAACCCTTATCTAATCTACAATCATCAAAAAC ‐3′
	*VEGF Region 2*	Forward: 5′‐ TTTTGAGGTYGTGGATTTTGGTAAGGGGTTTAG‐3′ Reverse: 5′‐TACAACTCRACTACCCCAAACCTCTAC
	*VEGF Region 3*	Forward: 5′‐ TTTATTAGTTYGGGAGTTTGTGTTTTGGGATTTG‐3′ Reverse:5‐AATAATTTAAAAAAATAAAAAACCAACCTCCTCAAACC‐3′
	*VEGF Region 4*	Forward: 5′‐ TAGTAGAYGAAAGAGGTATTAAGAGTTTTAG‐3′ Reverse: 5′‐ CTCTAACRATCACCCCCAAAAACAAAATCAAATCAAC‐3′
ChIP‐qPCR	*VEGF*	Forward: 5′‐CTTCAGTTCCCTGGCAACATCTC‐3′
		Reverse: 5′‐AGAGAATTTGGCACCAAATTTGT‐3′

### Western blot analysis

In Western blot experiments, five independent samples for each experimental condition, each containing two retinas from two different mice either WT or AQP4 KO, were used. Retinal samples were processed as previously reported 34 obtaining supernatants containing cytosolic proteins (used to detect VEGF, HIF‐1α, iNOS and nitrite production) or membrane proteins (used to detect AQP4). Nuclear proteins were obtained using a commercially available kit (Nuclear Extraction Kit; Abcam, Cambridge, UK). Protein concentration was determined using a fluorometer (Qubit; Invitrogen). Equal amounts (30 μg) of proteins were separated by SDS/PAGE and transferred onto PVDF membranes (Bio‐Rad Laboratories). β‐actin or histone H3 was used as loading controls. The blots were blocked in 3% skim milk for 1 hr at room temperature (RT) and then incubated overnight at 4°C with primary antibodies, followed by 1‐hr incubation at RT with secondary antibodies. Key features of the antibodies are summarized in Table 2. Blots were developed with an enhanced chemiluminescence reagent (Millipore, Billerica, MA, USA). The optical density of the bands was normalized to the level of β‐actin or histone H3 as specified. All experiments were run in duplicate. After statistical analysis, data from the different experiments were plotted and averaged on the same graph.

**Table 2:** Primary and secondary antibodies used in Western blot

Antibody	Dilution	Source	Catalogue
Goat polyclonal anti‐AQP4	1:300	Santa Cruz Biotechnology	sc‐9888
Rabbit polyclonal anti‐VEGF	1:200	Santa Cruz Biotechnology	sc‐507
Rabbit polyclonal anti‐HIF‐1α	1:200	Santa Cruz Biotechnology	sc‐10790
Rabbit polyclonal anti‐iNOS	1:200	Santa Cruz Biotechnology	sc‐8310
Mouse monoclonal anti‐β‐actin	1:2500	Sigma‐Aldrich	A2228
Rabbit polyclonal anti‐histone H3	1:5000	Abcam	ab21054
Rabbit anti‐goat IgG horseradish peroxidase‐labelled	1:5000	Santa Cruz Biotechnology	sc‐2768
Mouse anti‐rabbit IgG horseradish peroxidase‐labelled	1:5000	Santa Cruz Biotechnology	sc‐2357
Rabbit anti‐mouse IgG horseradish peroxidase‐labelled	1:25,000	Sigma‐Aldrich	A9044

### Enzyme‐linked immunosorbent assay

VEGF levels were measured in cytosolic protein‐containing supernatants used in Western blot analysis using a commercially available kit (R&D Systems, Minneapolis, MN, USA). VEGF levels were evaluated spectrophotometrically (Microplate Reader 680 XR; Bio‐Rad Laboratories). Data were expressed as picogram VEGF/mg protein. All experiments were run in duplicate. After statistical analysis, data from the different experiments were plotted and averaged in the same graph.

### Measurement of nitrite formation

The production of nitrite that indirectly detects total NO amount (μM) was evaluated spectrophotometrically (Microplate Reader 680 XR; Bio‐Rad Laboratories) by a Griess reaction kit (Enzo Life Sciences, Plymouth Meeting, PA, USA) in cytosolic protein‐containing supernatants used in Western blot analysis. All experiments were run in duplicate. After statistical analysis, data from the different experiments were plotted and averaged in the same graph.

### Immunohistochemistry and quantitative analysis

The retinal vasculature was visualized by immunohistochemistry on retinal whole mounts in line with previous works 22. Briefly, the retinal whole mounts were freeze‐thawed and incubated overnight at 4°C in fluorescein labelled isolectin B4 (1:200; Vector Laboratories, Burlingmae, CA, USA). Retinal whole mounts were examined for changes in their vascular pattern with a microscope equipped with epifluorescence (Eclipse Ni‐E; Nikon, Amsterdam, The Netherlands). Images of the retinal vasculature were acquired through a digital photocamera (DS‐Fi1c camera; Nikon) and processed using an image‐editing software (Adobe Photoshop; Adobe Systems, Inc., Mountain View, CA, USA) to create whole retina montages in which the extent of the avascular area and the total area of pre‐retinal neovascular tufts were measured according to previous quantification studies 34. For each experimental condition, quantitative data originated from six retinas from six different mice either WT or AQP4 KO.

### Electroretinography

Retinal function was examined with scotopic full‐field ERG as previously reported 35. After dark‐adaptation overnight, ERG responses were recorded from both eyes together using silver/silver chloride ring electrodes inserted under the lower eyelids, forehead reference electrode, and ground electrode in the tail. Pupils were fully dilated using 0.5% atropine. The cornea was intermittently irrigated with saline solution to maintain the baseline recording. Body temperature was maintained at 38°C by a heating pad. Mixed (rod and cone) responses were evoked by flashes of different light intensities ranging from −3.4 to 1 log cd‐s/m^2^ generated through a Ganzfeld stimulator (Biomedica Mangoni, Pisa, Italy). The electrodes were connected to a two‐channel amplifier. Signals were amplified at 1000 gain and bandpass filtered between 0.2 and 500 Hz before being digitized at 5 kHz rate with a data acquisition device (Biomedica Mangoni). The ERG waveforms were examined primarily for amplitude information, and the data (pooled and reported as mean amplitudes ± S.E.M. in μV) were graphed to determine any gross changes in the intensity‐response function for that eye. Intensity‐response functions of the b‐wave were fit to a modified Naka‐Rushton function 36 V(I) = V0 + (Vmax·I^n^)/(I^n^ + k^n^). In this equation, V is the amplitude of the b‐wave (in μV), I is the stimulus intensity (in log cd‐s/m^2^), V0 is the non‐zero baseline effect, Vmax is the saturated amplitude of the b‐wave (in μV), k is the stimulus intensity that evokes a b‐wave of half‐maximum amplitude (in log cd‐s/m^2^), *n*, which was constrained to unity, is a dimensionless constant controlling the slope of the function and represents the degree of heterogeneity of retinal sensitivity. To demonstrate oscillatory potentials (OPs), ERG responses were digitally filtered with bandpass filter between 65 and 300 Hz to eliminate the a‐ and b‐wave interference and to avoid the 60 Hz line noise. Of the five OPs that can generally be isolated from the mouse ERG, only OP2, OP3 and OP4 were analysed as OP1, and OP5 could not be accurately measured at PD17 37. For each OP, the trough‐to‐peak amplitude was measured and the amplitudes of each wavelet were added to create the sum OPs (SOPs = OP2 + OP3 + OP4; 38). Mean amplitudes of ERG responses were plotted as a function of increasing light intensities. For each experimental condition, ERG analysis was performed on six mice either WT or AQP4 KO.

### Analysis of DNA methylation

For each experimental condition, genomic DNA was extracted from the retinas of four mice either WT or AQP4 KO mice, killed at PD12 or PD17, using a kit (DNeasy Blood & Tissue Kit; Qiagen). Bisulphite reaction was performed (EpiTect Bisulfite Kit; Qiagen) to deaminate unmethylated cytosine to produce uracil in DNA. Purified bisulphite‐treated DNA was quantified using a spectrophotometer (NanoDrop 2000; Thermo Fisher Scientific, Waltham, MA, USA). To search for CpG sites, the promoter of mouse VEGF (GenBank: U41383.1) was analysed by MethPrimer 39. The online available ZymoResearch Software (http://www.zymoresearch.com/tools/bisulfite-primer-seeker) was used to design primers for bisulphite sequencing. Their sequences are shown in Table 1. Fifty nanogram of bisulphite treated and purified DNA was PCR amplified using these primers and High Fidelity Taq DNA Polymerase (Life Technologies, Carlsbad, CA, USA). PCR products were analysed by 2% agarose gel, and amplicons were eluted by QIAEX II Gel Extraction Kit (Qiagen) and sequenced. FinchTV version 1.5.0 (free program from Geospiza, inc.) was used to analyse sequences and to detect double peaks (C/T). All unmethylated cytosines (C) were sequenced as thymine (T) and the presence of a C‐peak indicated the presence of 5mC in the genome. The presence of both C‐ and T‐peaks indicated partial methylation, and the height of C peaks was proportional to methylation frequency. The methylation frequency of the CpG site in the HBS was quantified in retinas at PD12. The Combined Bisulfite Restriction Analysis (COBRA) of the HBS was performed as reported previously 40. Briefly, 300 ng of PCR products containing the HBS was digested overnight at 37°C with HPYcH4IV restriction enzyme (New England Biolabs, Ipswich, Massachusetts, USA), which is selective for methylated HBS. Restriction fragments were analysed by 2.5% agarose gel, calculating the % of methylated sequences with ImageJ software (http://imagej.nih.gov/ij/; provided in the public domain by the National Institutes of Health, Bethesda, MD, USA). Methylation frequencies of the CpG1 site (inside the HBS) and three CpG sites (CpG2, 3 and 4) proximal to the HBS were measured by the cloning and sequencing approach, performed as follows. Eight post‐bisulphite PCR products were equimolar pooled (four WT and four AQP4 KO) and cloned into pcR2.1 Topo‐TA vector using Top10 competent cells (Life Technologies). Independent clones were fully sequenced and aligned to reveal C (methylated C in genome) or T (demethylated C in genome) in the CpG1 of the HBS (TA***C***GTGGG) and in CpG2‐4 in the same PCR product.

### HIF‐1α‐specific chromatin immunoprecipitation and HBS‐specific qPCR (ChIP‐qPCR)

#### Chromatin immunoprecipitation (ChIP)

ChIP was performed using the MAGnify Chromatin Immunoprecipitation System kit (Thermo Fisher Scientific, Life Thechnologies) with significant modification relative to the amount of starting material and chromatin fragmentation conditions. Each sample was composed of two retinas from the same mouse. Retinas were explanted at PD12 after 1 and 6 hrs of hypoxia and at PD17, immediately flash frozen in liquid nitrogen and stored at −80°C until the chromatin preparation. Samples were thawed on ice and homogenized with a sterile 1‐ml syringe and a 1.5‐inch 20G needle in 75 μl of ice‐cold D‐PBS. Sixty microlitre of room temperature D‐PBS was added to the homogenized tissue off the ice. To fix the samples and crosslink the chromatin, 4 μl of 37% formaldehyde was added and samples were incubated for 10 min. at RT, swirling the tube every 2 min. The crosslink was quenched with 17 μl of 1.25 M glycine for 5 min. at RT, and cells were harvested by centrifugation (5000× *g* for 3 min. at 4°C), washed twice with 150 μl of ice‐cold D‐PBS. The pellet was extracted on ice with 80 μl of lysis buffer and protease inhibitor provided in the kit. Chromatin shearing was performed with Vibra‐Cell Sonics VC‐130 for 10 cycles on ice (20 sec. of sonication at 25% amplitude followed by 20 sec. rest on ice) to obtain an average smear of 1 Kbp. Chromatin shearing was checked by agarose gel electrophoresis. Immunoprecipitation was performed using 10 μl of Dynabeads Protein A/G, 1 μl of rabbit polyclonal Anti‐HIF‐1α ChIP Grade antibody (ab2185; Abcam) or control rabbit IgG provided in the kit and 15 μl of sheared chromatin with 10 r.p.m. rotation at 4°C over night. DNA from 1.5 μl of input chromatin (10% of immunoprecipitated chromatin) was purified and used in the subsequent qPCR as input sample. For each time‐point, the chromatin of 3 WT and 3 AQP4 KO mice was individually immunoprecipitated.

#### HBS specific qPCR

Real‐Time qPCR was performed using Power‐UP SybrGreen and the StepOne system (Thermo Fisher Scientific, Life Thechnologies). Two microlitre of eluted DNA, from HIF‐1α ChIP and control IgG ChIP, and 2 μl of input DNA, were amplified using primers mapping upstream and downstream of the HBS in the mouse VEGF promoter (see Table 1). The percentage input method was used to measure the HIF‐1α binding to the HBS. Briefly, starting from the Ct obtained from amplification plots relative to the input, to the control IgG ChIP and to the HIF‐1α ChIP, the IgG‐normalized input percentage was calculated (input percentage relative to HIF‐1α ChIP—input percentage relative to IgG ChIP). The results were expressed as means ± S.E.M. of IgG‐normalized input percentage.

### Statistics

All data were analysed by the Shapiro–Wilk test to verify their normal distribution. Statistical significance was evaluated using unpaired Student's *t*‐test, One‐way analysis of variance (anova) followed by Newman–Keuls’ multiple comparison post‐test, or two‐way anova followed by Bonferroni's post‐test, as appropriate. The results were expressed as means ± S.E.M. of the indicated *n* values. Prism 5 (GraphPad Software, Inc., La Jolla, CA, USA) was used to analyse data. Differences with *P* < 0.05 were considered statistically significant.

## Results

### Retinal levels of AQP4, VEGF, HIF‐1α, iNOS and NO

**Figure 1:**
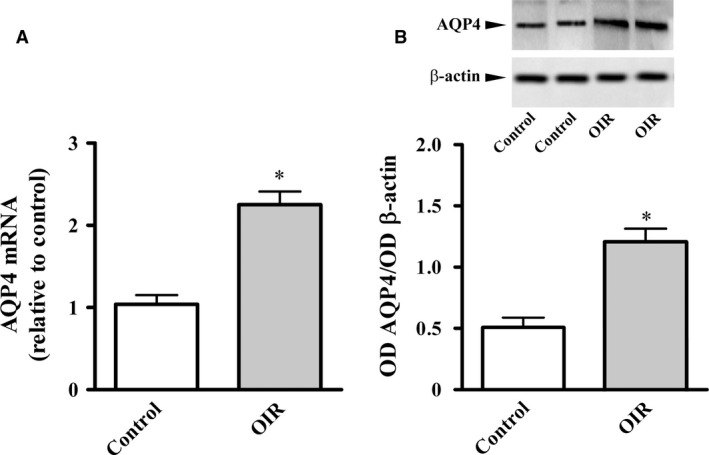
Retinal levels of AQP4 in WT mice at PD17. (**A**) AQP4 mRNA, measured by qPCR, was significantly higher in OIR than in normoxic controls. Western blot and densitometric analysis (**B**) confirmed qPCR data. Data are presented as mean ± S.E.M. (*n* = 5, *P < 0.01 *versus* control. Unpaired Student's *t*‐test).

In line with previous findings 8, we found that AQP4 was upregulated by 5 days of relative hypoxia (PD17). Levels of AQP4 mRNA and proteins were about 115% and 126% (*P* < 0.01) higher than in normoxic controls (Fig. 1).

**Figure 2:**
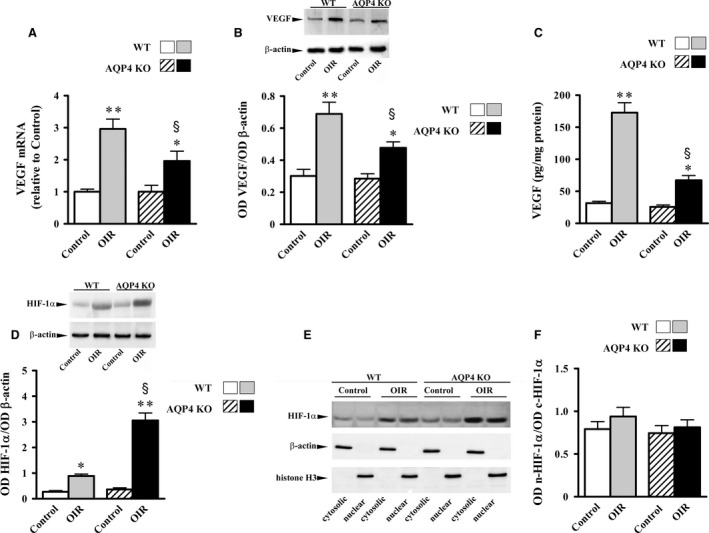
Retinal levels of VEGF and HIF‐1α in WT and AQP4 KO mice at PD17. (**A**) VEGF mRNA, measured by qPCR, was significantly higher in OIR than in normoxic controls in both WT and AQP4 KO mice. In normoxic controls, VEGF mRNA did not differ between WT and AQP4 KO mice, while in OIR, VEGF mRNA was significantly lower in AQP4 KO than in WT mice. Western blot and densitometric analysis (**B**), as well as ELISA (**C**), confirmed qPCR data. (**D**) HIF‐1α levels, measured by Western blot and densitometric analysis, were significantly higher in OIR than in normoxic controls in both WT and AQP4 KO mice. In normoxic controls, HIF‐1α levels did not differ between WT and AQP4 KO mice, while in OIR, HIF‐1α levels were significantly higher in AQP4 KO than in WT mice. (**E, F**) The ratios between nuclear and cytosolic HIF‐1α levels were evaluated by Western blot (**E**) and densitometric analysis (**F**). In both WT and AQP4 KO mice, either controls or OIR, similar ratios between nuclear and cytosolic levels of HIF‐1α were measured. Data are presented as mean ± S.E.M. (*n* = 5, *P < 0.01 and **P < 0.001 *versus* respective control; ^§^ *P* < 0.01 *versus* OIR WT. One‐way anova followed by Newman‐Keuls’ multiple comparison post‐test). White bars, control WT; grey bars, OIR WT; dashed bars, control AQP4 KO; black bars, OIR AQP4 KO; n, nuclear; c, cytosolic.

**Figure 3:**
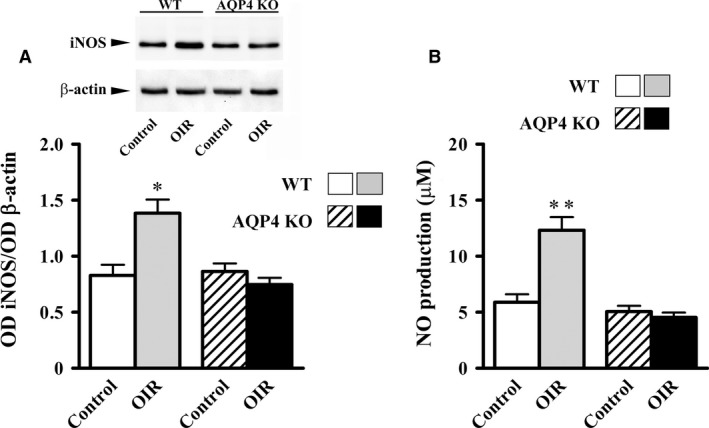
Retinal levels of iNOS and NO in WT and AQP4 KO mice at PD17. As evaluated by Western blot and densitometric analysis (**A**), iNOS levels in OIR were significantly higher than in normoxic controls in WT but not in AQP4 KO mice. Consistently, as evaluated by the Griess method (**B**), NO production in OIR was significantly higher than in normoxic controls in WT but not in AQP4 KO mice. Data are presented as mean ± S.E.M. (*n* = 5,*P < 0.01 and **P < 0.001 *versus* respective control. One‐way anova followed by Newman‐Keuls’ multiple comparison post test). White bars, control WT; grey bars, OIR WT; dashed bars, control AQP4 KO; black bars, OIR AQP4 KO.

Whether AQP4 deletion affects retinal levels of VEGF, HIF‐1α, iNOS and NO in response to hypoxia was investigated. As shown in Figures 2 and 3, normoxic levels of VEGF mRNA (Fig. 2A), VEGF proteins (Fig. 2B and C), HIF‐1α proteins (Fig. 2D), iNOS proteins (Fig. 3A) and NO production (Fig. 3B) did not differ between WT and AQP4 KO mice. Hypoxia increased VEGF mRNA by about 200% in respect to normoxia in WT mice (*P* < 0.001) and by about 96% in AQP4 KO mice (*P* < 0.01). VEGF proteins as evaluated by Western blot were drastically increased by hypoxia (about 130% in WT mice, *P* < 0.001 and about 70% in AQP4 KO mice, *P* < 0.01). As evaluated by ELISA, hypoxia increased VEGF levels (about 450% in WT mice, *P* < 0.001 and about 160% in AQP4 KO mice, *P* < 0.01). Figure 2D shows that hypoxia increased HIF‐1α proteins by about 225% in WT mice (*P* < 0.01) and by about 735% in AQP4 KO mice (*P* < 0.001). We checked whether increased HIF‐1α accumulation in the absence of AQP4 might depend on an impaired migration of HIF‐1α from the cytosol to the nucleus. Representative blots depicting cytosolic and nuclear HIF‐1α are shown in Figure 2E. The densitometric analysis of Figure 2F demonstrates that the ratios between nuclear and cytosolic levels of HIF‐1α did not differ between WT and AQP4 KO mice. Measurements of iNOS proteins and NO production in response to hypoxia shown in Figure 3 demonstrate that iNOS increased by about 70% in WT mice (*P* < 0.01), whereas it was not influenced by hypoxia in AQP4 KO mice. Consistently, hypoxia increased NO production by about 110% (*P* < 0.001) in WT mice, while no effects were found in AQP4 KO mice.

### Retinal vasculature

**Figure 4:**
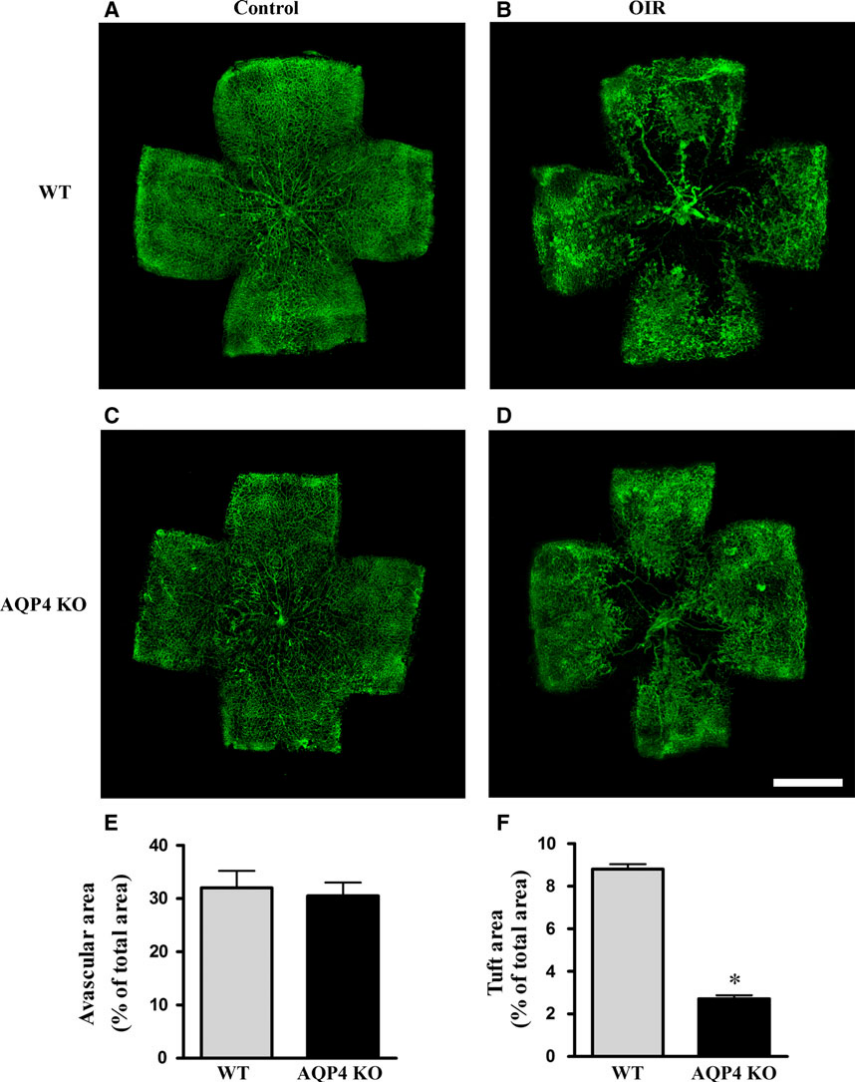
Representative images of retinal flat mounts from WT and AQP4 KO mice at PD17. Retinal vessels were identified with fluorescein labelled isolectin B4. The vascular pattern of control retinas did not differ between WT (**A**) and AQP4 KO (**C**) mice. In OIR WT (**B**) and AQP4 KO (**D**) mice, a large avascular area could be seen in the central retina. In contrast to what was found in WT, AQP4 KO mice exhibited only rare neovascular tufts. Scale bar: 1 mm. The extent of both the avascular area (**E**) and the tuft area (**F**) were quantitatively evaluated in WT and AQP4 KO mice. AQP4 deletion did not affect the extent of the avascular area while it reduced the neovascular tuft area. Data are presented as means ± S.E.M. (*n* = 6, *P < 0.001 *versus* WT. Unpaired Student's *t*‐test).

The vascular pattern of normoxic retinas did not differ between WT and AQP4 KO mice (Fig. 4A and C). In WT mice, the vascular pattern of the hypoxic retina was consistent with previous results 22 and was characterized by a large avascular area in the central retina and an excessive regrowth of abnormal superficial vessels in the mid‐peripheral retina (Fig. 4B). In AQP4 KO mice, instead, the superficial plexus exhibited only rare neovascular tufts without any apparent difference in the extent of the avascular area (Fig. 4D). The quantitative analysis confirmed that AQP4 deletion did not affect the avascular area (Fig. 4E), whereas it reduced significantly the area occupied by neovascular tufts (about 80%, *P* < 0.001; Fig. 4F).

### ERG responses

**Figure 5:**
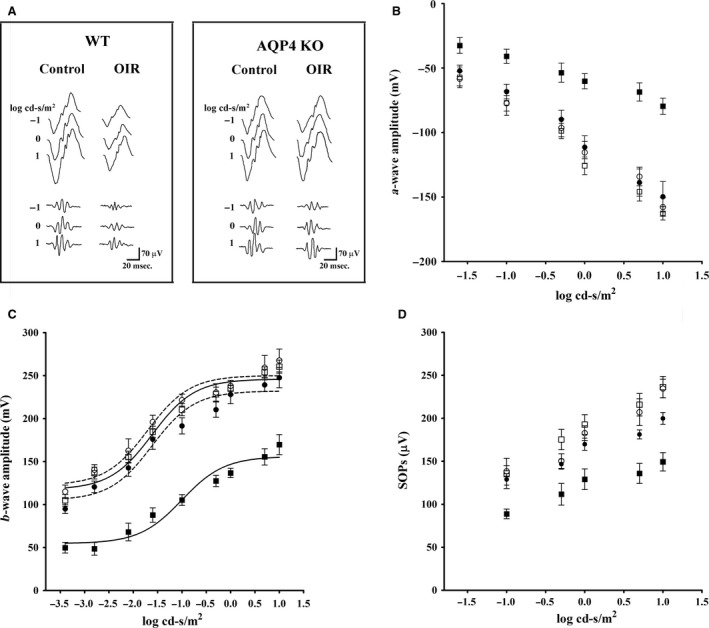
ERG responses in WT and AQP4 KO mice at PD17. (**A**) Representative ERG waveforms in both WT and AQP4 KO mice, either controls or OIR, recorded at light intensities of ‐1, 0 and 1 log cd‐s/m2. (**B–D**) a‐wave, b‐wave and SOP amplitudes in normoxic controls (white symbols) and OIR (black symbols) either WT (squares) or AQP4 KO (circles) at increasing light intensities. AQP4 deletion prevented the OIR‐induced reduction in a‐waves, b‐waves and SOPs that characterize OIR. Data are presented as mean ± S.E.M. (*n* = 6 mice, *P* < 0.001 *versus* OIR WT. Two‐way anova followed by Bonferroni's post‐test).

Representative mixed a‐, b‐waves and OPs recorded in both normoxic and hypoxic conditions in WT or AQP4 KO mice are shown in Figure 5A. Increased a‐ and b‐wave amplitudes with increasing stimulus intensity were observed (Fig. 5B and C). A clear a‐wave developed at a light intensity of approximately −1.6 log cd‐s/m^2^. In normoxia, WT and AQP4 KO mice had comparable ERG responses. In response to hypoxia, WT mice displayed reduced a‐ and b‐wave amplitudes in respect to normoxia at all light intensities (*P* < 0.001). In contrast, AQP4 KO mice displayed an a‐ and b‐wave amplitudes which did not differ from those measured in normoxia. The amplitudes of b‐waves obtained over varying flash light intensities were fitted using the Naka‐Rushton equation to evaluate the post‐receptor response amplitude (Vmax) and the retinal sensitivity (k). As shown in Table 3, in OIR WT mice, the values of Vmax and k were significantly lower than in normoxia (*P* < 0.001), whereas in AQP4 KO mice, the values of Vmax and k were not significantly different from those in normoxia. As shown in Figure 5D, summed OP amplitudes (SOPs) were averaged as a function of increasing light intensities. In normoxia, both WT and AQP4 KO mice displayed increased SOPs with increasing stimulus intensity. In hypoxia, WT mice were characterized by SOPs reduction at all light intensities (*P* < 0. 001). In AQP4 KO mice, SOPs did not differ significantly from those measured in normoxia.

**Table 3:** Parameters obtained from b‐wave amplitude using the Naka‐Rushton function

	WT		AQP4 KO	
	Control	OIR	Control	OIR
Vmax (μV)	246.00 ± 4.05	156.00 ± 5.47^a^	250.10 ± 5.4	232.10 ± 5.11
K (log cd‐s/m^2^)	−1.60 ± 0.11	−1.00 ± 0.15^a^	−1.71 ± 0.14	−1.61 ± 0.13

a *P* < 0.001 *versus* control.

### DNA methylation analysis of the *VEGF* gene promoter: focus on the HBS

**Figure 6:**
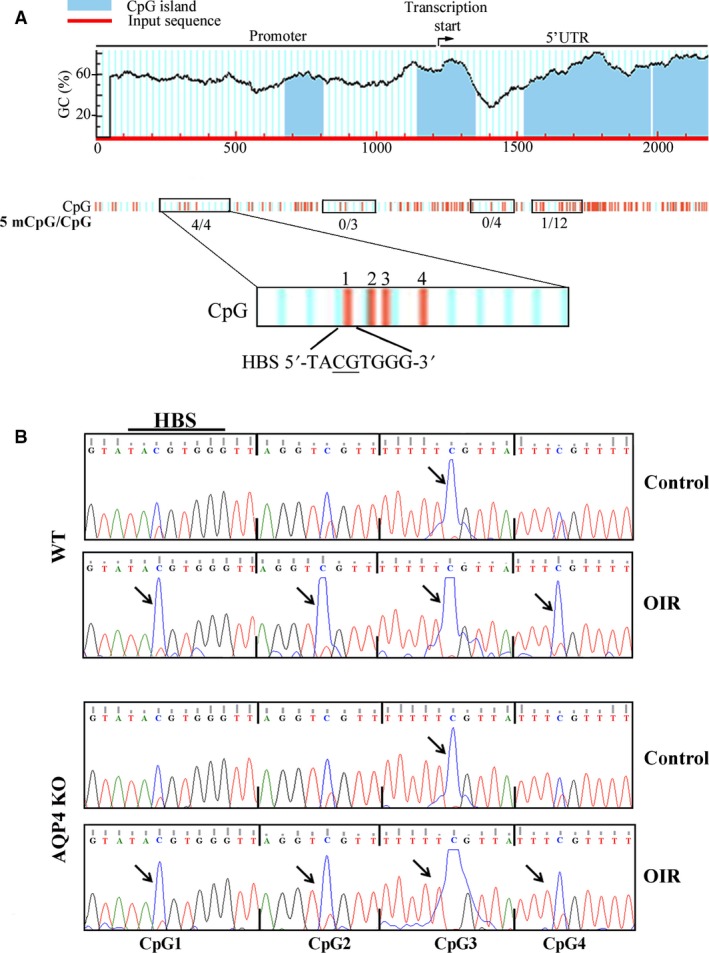
CpG site methylation analysis in the VEGF promoter in WT and AQP4 KO mice at PD17. (**A**) CpG sites are highlighted by vertical red lines. The position of the HBS is reported and the relative CpG site underlined. Methylation status of 21 CpG sites in four DNA region (boxed) of the VEGF promoter was determined by direct PCR bisulphite sequencing. All four CpG sites were only found to be methylated in the region containing the HBS. (**B**) Representative chromatograms of PCR bisulphite sequencing of the boxed DNA region reported in panel **A**. C/T double peaks in the CpG sites in the DNA region containing the HBS (CpG sites 1‐4) are shown. Normoxic controls, either WT or AQP4 KO, were characterized by the complete methylation of CpG3 (blue peak, arrow). In OIR, either WT or AQP4 KO, blue peaks (5 methyl CpG, arrows) were increased in a similar fashion. The analysis was performed on four independent samples for each experimental condition, each containing one retina.

We investigated in retinas from AQP4 KO mice whether the lower VEGF accumulation in response to hypoxia might be associated with changes in the CpG methylation status in the VEGF gene promoter. In a first set of experiments, direct PCR bisulphite sequencing was used to analyse the methylation status of 23 CpG sites in 4 DNA regions belonging to the VEGF promoter and the 5′ UTR. In particular, we analysed 11 single CpG sites, including the CpG site contained in the HBS 41 (CpG1), as well as 12 CpG sites contained in a 5′UTR CpG island. Figure 6A shows that after 5 days of hypoxia (PD17), 4 CpG sites (CpG1 in the HBS and three other nearby CpG sites, CpG2, CpG3 and CpG4) were methylated in both WT and AQP4 KO mice, while the CpG sites in the remaining DNA regions were almost completely demethylated in the two strains. In Figure 6B, the chromatograms that are representative of the methylation status of CpG1‐4 are shown. It can be noticed that the methylation status of CpG1‐4 did not differ between WT and AQP4 KO mice with CpG3 that was methylated in both normoxia and hypoxia, while CpG1, 2 and 4 that were demethylated in normoxia, but methylated in hypoxia. This finding demonstrates that at PD17, there was no difference in the methylation status between WT and AQP4 KO mice although, in the absence of AQP4, mRNA levels of VEGF were lower than in its presence.

**Figure 7:**
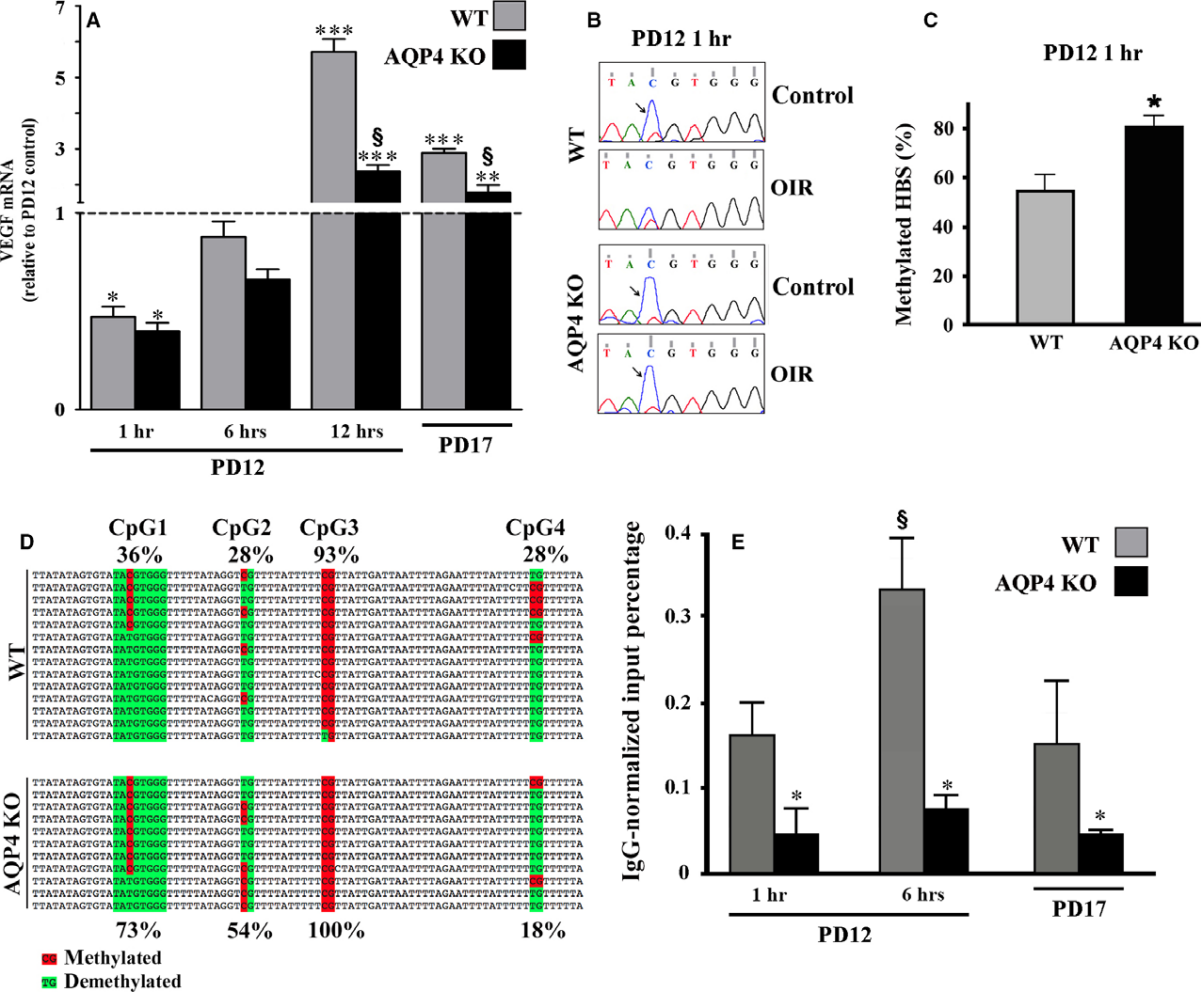
Analysis of VEGF mRNA levels, CpG methylation status and physical interaction between HIF‐1α and HBS. (**A**) VEGF mRNA levels were evaluated by qPCR at different times after hypoxia. At 1 hr, hypoxic levels of VEGF mRNA were significantly lower than in normoxia to then increase at 6 hrs with a peak at 12 hrs followed by a drastic reduction at PD17. The absence of AQP4 reduced VEGF mRNA levels already after 12 hrs of hypoxia. (*P < 0.05, **P < 0.01, ***P < 0.001 *versus* respective control; ^§^ *P* < 0.001 *versus* WT; anova). (**B**) Representative chromatograms of PCR bisulphite sequencing and relative C/T double peaks in the CpG sites of the HBS. Blue peaks (5 methyl CpG, arrows) indicate that in normoxic controls, the CpG site was methylated in retinas of both WT and AQP4 KO mice, while in OIR, the CpG site became demethylated in WT, but not in AQP4 KO mice. (**C**) The COBRA analysis confirmed that the methylation frequency of the CpG site in the HBS was significantly higher in AQP4 KO than in WT mice (*P < 0.05 *versus* WT, unpaired Student's *t*‐test). (**D**) The cloning and the sequencing of bisulphite‐treated DNA relative to four CpG sites (CpG1‐4) showed that the methylation frequency of the CpG1 site in the HBS was about twofold higher in AQP4 KO than in WT mice, similarly for CpG2. CpG3 was completely methylated while CpG4 was strongly demethylated, both in a similar fashion between WT and AQP4 KO mice. (**E**) HIF‐1α chromatin immunoprecipitation and HBS‐specific qPCR (ChIP‐qPCR) were performed on WT and AQP4 KO mice. In WT mice, the physical interaction between HIF‐1α and the HBS starts quickly and reaches a peak after 6 hrs, while in AQP4 KO mice, the interaction is always very low and appears to be insensitive to hypoxia (*P < 0.05 *versus* relative WT; ^§^ *P* < 0.05 6 hrs *versus* 1 hr and 6 hrs *versus* PD17 in WT, anova).

Considering that HBS demethylation is required to allow HIF‐1α interaction with the HBS 27, that this interaction should precede VEGF production 41 and that retinal levels of HIF‐1α and VEGF are maximally upregulated in response to hypoxia over the first hours after the hypoxia onset 23, we can reasonably assume that in the OIR model, the interaction between HIF‐1 and the HBS should take place rapidly during the hypoxic response. In a first set of experiments, we evaluated VEGF expression at different times after the beginning of hypoxia in WT and AQP4 KO mice. We found that at 1 hr of hypoxia, the levels of VEGF mRNA were significantly lower than in normoxia. VEGF mRNA reached normoxic levels after 6 hrs of hypoxia to drastically increase at 12 hrs after then they were reduced by about 50%. We found that compared to the WT the absence of AQP4 had already reduced VEGF mRNA levels after 12 hrs of hypoxia when the VEGF accumulation was maximal (Fig. 7A). To test the possibility that hypoxia rapidly influences the methylation status of the HBS and to evaluate the effect of AQP4 absence, we investigated the methylation status of CpG1 after 1 hr of hypoxia. As shown in Figure 7B, in response to 1 hr of hypoxia, CpG1 was indeed demethlyated in WT mice, whereas it was methylated in AQP4 KO mice similarly to what found in both WT and AQP4 KO mice under normoxia. The COBRA analysis revealed that after 1 hr of hypoxia, the methylation frequency of CpG1 was about 30% lower in WT than in AQP4 KO mice (*P* < 0.05; Fig. 7C). An additional quantification of the methylation frequency of CpG1‐4 in the VEGF promoter was performed using the most accurate technique of cloning and sequencing of bisulphite‐treated DNA. Figure 7D shows that the methylation frequency of CpG1 in the HBS was about twofold lower in WT than in AQP4 KO mice (36% in WT *versus* 73% in AQP4 KO mice). Similarly, the methylation frequency of CpG2 is about 28% in WT and 54% in AQP4 KO mice. In contrast, CpG3 was almost completely methylated, and CpG4 strongly demethylated, in both WT and AQP4 KO mice without any difference between the two strains.

### Analysis of physical interaction between HIF‐1α and the HBS of the *VEGF* gene promoter in OIR

To test whether the different methylation frequency of the CpG1 in the HBS measured in hypoxia was associated to a different physical interaction between HIF‐1α and the HBS, retinas of WT and AQP4 KO mice were analysed at PD12 after 1 and 6 hrs of hypoxia and at PD17 by HIF‐1α‐specific chromatin immunoprecipitation and HBS‐specific qPCR (ChIP‐qPCR). Due to the very low level of HIF‐1α measured in normoxia (Fig. 2D) and due to the almost absent interaction between HIF‐1α and the HBS occurring in normoxia already reported in the literature 42, the ChIP‐qPCR analysis was here restricted to the hypoxic condition by comparing WT with AQP4 KO mice. As shown in Figure 7E, in WT mice, the interaction between HIF‐1α and the HBS started quickly (in the first hour) and peaked after 6 hrs of hypoxia, whereas it had returned to levels measured after 1 hr of hypoxia at PD17. In AQP4 KO mice, the interaction between HIF‐1α and the HBS was lower than in WT mice: three times after 1 hr of hypoxia or at PD17 and six times after 6 hrs of hypoxia.

## Discussion

This is the first demonstration that hypoxia induces HBS demethylation at level of VEGF promoter *in vivo* and that AQP4 deletion prevents this process. The lack of HBS demethylation would limit the accessibility of HIF‐1 to the *VEGF* gene promoter thus reducing the hypoxia‐induced *VEGF* gene transactivation and VEGF accumulation in response to hypoxia therefore preventing the retinal damage including pathological neovascularization and retinal dysfunction.

### Mechanisms underlying lower VEGF accumulation in AQP4 KO mice: HIF‐1α accumulation and translocation

As shown by the present results, in AQP4 KO mice, hypoxic conditions are coupled to lower retinal levels of VEGF as compared to WT mice thus indicating that AQP4 may have a detrimental role during ischaemic/hypoxic conditions of the retina. This is in line with the fact that AQP4 is overexpressed in the OIR model as demonstrated here, a finding which is consistent with previous results in rodent models of neovascular pathologies of the retina 7, 8, 9, 10, 11, 14, 16 and with the observation that AQP4 deletion ameliorates the pathological signs of retinal ischaemia 6.

In search for mechanisms underlying this reduced VEGF levels in response to hypoxia, we have first checked whether HIF‐1α levels may be in parallel reduced by the absence of AQP4 as VEGF and HIF‐1α levels are strictly interconnected 43. However, HIF‐1α is instead dramatically increased in response to hypoxia in AQP4 KO mice. One possibility to explain HIF‐1α overproduction in the absence of AQP4 would be to postulate that AQP4 deletion prevents NO accumulation in response to hypoxia, an event that is known to limit HIF‐1α stabilization in hypoxic conditions 24. As shown here, in the absence of AQP4, neither iNOS expression nor NO production is influenced by hypoxia thus explaining why HIF‐1α stabilization is out of control in the absence of NO overproduction. On the other hand, the lack of NO upregulation may directly cause reduced VEGF levels as NO is known to induce VEGF expression 44. In the OIR model, for instance, inhibition of iNOS has been observed to reduce retinal VEGF 45, 46. Conversely, VEGF may act as an upstream regulator of iNOS 47, thus indicating that reduced VEGF levels may cause reduced iNOS expression in response to hypoxia. Thus, the complex relationship between iNOS and VEGF is characterized by reciprocal interactions in which AQP4 may play a role, although its exact contribution and the mechanisms involved remain to be investigated.

An additional possibility to explain the reduced VEGF accumulation in response to hypoxia is that the translocation of HIF‐1α from the cytosol to the nucleus would be impaired in the absence of AQP4, thus limiting the availability of HIF‐1 to the HBS in the *VEGF* gene promoter and reducing HIF‐1 transcriptional activity. In this respect, proteins such as HIF hydroxylases may sequester HIF‐1α into the cytoplasm thus preventing its nuclear migration and reducing HIF‐1 transcriptional activity 48. This possibility, however, has been denied by the fact that the ratio between nuclear and cytoplasmic levels of HIF‐1α is not influenced by AQP4 deletion although in the presence of a reduced HIF‐1 transcriptional activity.

### Mechanisms underlying lower VEGF accumulation in AQP4 KO mice: HBS demethylation and HIF‐1α binding

**Figure 8:**
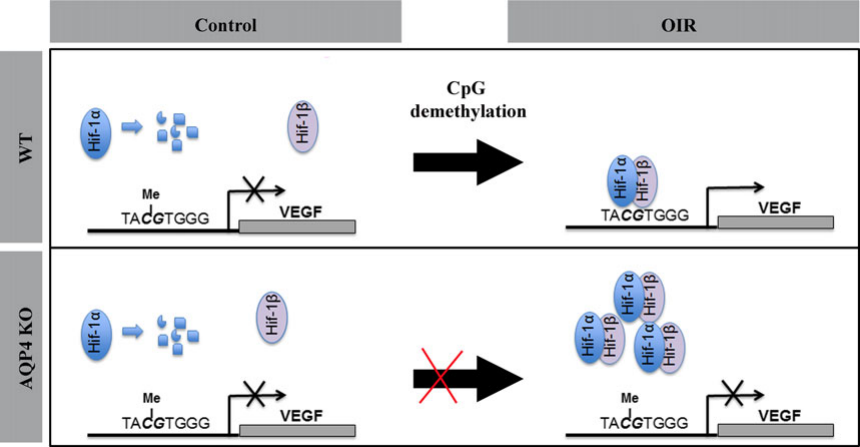
Schematic diagram depicting the methylation status of the HBS in the VEGF promoter in both WT and AQP4 KO mice, the HIF‐1 binding and the relative VEGF transactivation. In both WT and AQP4 KO mice under normoxia, HIF‐1α is degradated and no VEGF transactivation occurs. In WT mice HIF‐1α is stabilized, interacts with HIF‐1β and binds the demethylated HBS localized in the VEGF promoter. In AQP4 KO mice, despite high levels of HIF‐1α, the absence of HBS demethylation prevents the HIF‐1 binding and the relative VEGF transactivation. Our assumption is that the absence of AQP4 reduces HBS demethylation limiting the accessibility of HIF‐1 to the *VEGF* gene promoter, therefore reducing VEGF transcription and accumulation in response to hypoxia.

It is known that hypoxia induces CpG demethylation at the level of promoters of hypoxia‐regulated genes 49, 50 thus allowing the binding of transcription factors and gene transcription. For instance, HBS demethylation is a mandatory process to allow HIF‐1‐dependent erythropoietin gene expression 27. Little is known about the kinetic of CpG demethylation in the HBS and about the kinetic of the interaction with HIF‐1. Results in human neuroblastoma cells have demonstrated that the HBS in the VEGF promoter is demethylated after 24 hrs of hypoxia and that VEGF mRNA increases at 16 hrs of hypoxia strictly dependent on the enzymes involved in CpG demethylation 50 supporting the hypothesis that during the hypoxic response, a rapid CpG demethylation occurs at the HBS and that there is a tight correlation between hypoxia‐induced CpG demethylation and VEGF transactivation. In this respect, recent findings in rat pulmonary artery endothelial cells indicate that HIF‐1α binds the HBS within 1 hr of hypoxia, with a peak after 3 hrs 42. This result suggests that CpG demethylation at the HBS level, a mandatory process for HIF‐1 binding 27, occurs quickly in hypoxic conditions. More generally, there are also indications that the interaction between the VEGF promoter and hypoxia‐activated transcription factors occurs rapidly during the hypoxic response. For instance, phosphorylated STAT‐3 (signal transducer and activator of transcription 3, a transcription factor participating to hypoxia‐mediated VEGF transactivation) binds to the VEGF promoter after 30 min. of hypoxia in retinal endothelial cells 51. However, little is known about these mechanisms in the hypoxic retina. There is evidence that HIF‐1α is regulated over time after the beginning of hypoxia with a peak of stabilization occurring at 2 hrs 23. In addition, VEGF mRNA is constitutively expressed in the inner nuclear layer where it drastically increases 6 hrs after the onset of hypoxia and remains elevated for several days 23. This is also confirmed here by the demonstration that maximal accumulation of VEGF mRNA takes places between 6 and 12 hrs of hypoxia. As also shown by the present results, 1 hr of hypoxia induces HBS demethylation at the level of the VEGF promoter in line with the time course of retinal accumulation of HIF‐1α and VEGF mRNA reported during the hypoxic response 23. The additional evidence from the present results that AQP4 deletion drastically reduces VEGF expression already after 12 hrs of hypoxia is indicative of the fact that mechanisms of VEGF transactivation might be influenced by AQP4 absence already after the first hours of hypoxia. In this line, we found that AQP4 deletion prevents the HBS demethylation process. To provide direct evidence supporting the hypothesis that the observed change in the HBS methylation status may affect HIF‐1 accessibility to the VEGF promoter, therefore influencing retinal levels of VEGF, we checked the physical interaction between HIF‐1α and the HBS in the VEGF promoter. We show here for the first time in hypoxic retina that a physical interaction between HIF‐1α and the HBS occurs in the first hours of hypoxia and that this interaction is strongly affected by AQP4. The schematic diagram in Figure 8 shows the hypothetical sequence of events in normoxia and hypoxia in WT and AQP4 KO mice. We suggest that the absence of AQP4 reduces HBS demethylation limiting the accessibility of HIF‐1 to the *VEGF* gene promoter therefore reducing VEGF transcription and accumulation in response to hypoxia. As a consequence, neovascular tuft formation and ERG dysfunction, events downstream to VEGF upregulation in response to hypoxia, are prevented despite the hypoxic insult.

Taken together, the present results suggest a correlation between VEGF upregulation in response to hypoxia and HBS methylation status. AQP4 deletion would prevent VEGF upregulation presumably by interfering with hypoxia‐induced HBS demethylation process, thus reducing the physical interaction between HIF‐1 and the HBS. The additional observation that, in the absence of AQP4, iNOS, another HIF‐1 target gene, is not influenced by hypoxia suggests reduced demethylation processes as more general mechanisms of gene silencing through which AQP4 deletion may lead to decreased expression of HIF‐1 target genes in hypoxic conditions. How AQP4 regulates the mechanistic associations among VEGF, HIF‐1α and HBS demethylation is difficult to postulate as the mechanisms underlying the control of HBS demethylation process are still largely unknown 52. In this respect, understanding how a water channel may influence a site‐specific and hypoxia‐induced demethylation process will require further work and the use of different experimental models to exclude the possibility of off target effects due to the lack of *AQP4* gene. In conclusion, although with the difficulty of explaining the mechanistic relationships between AQP4 and VEGF and in the presence of mostly descriptive results, the present work lays the ground for future investigations for clarifying these issues.

## Conflict of interest

The authors have no conflicts of interest with the contents of this article.
